# Contribution of gut microbiomes and their metabolomes to the performance of Dorper and Tan sheep

**DOI:** 10.3389/fmicb.2022.1047744

**Published:** 2022-11-28

**Authors:** Yuhao Ma, Xue Yang, Guoying Hua, Xiaotian Deng, Tianlan Xia, Xinhai Li, Dengzhen Feng, Xuemei Deng

**Affiliations:** ^1^Key Laboratory of Animal Genetics, Breeding, and Reproduction of the Ministry of Agriculture and Beijing Key Laboratory of Animal Genetic Improvement, China Agricultural University, Beijing, China; ^2^Department of Animal Science and College of Agriculture, Ningxia University, Yinchuan, China

**Keywords:** microbiota, metabolomics, Dorper sheep, Tan sheep, individualized performance

## Abstract

**Background:**

Livestock is an excellent source of high nutritional value protein for humans; breeding livestock is focused on improving meat productivity and quality. Dorper sheep is a distinguished breed with an excellent growth performance, while Tan sheep is a Chinese local breed famous for its delicious meat. Several studies have demonstrated that the composition of gut microbiome and metabolome modulate host phenotype.

**Methods:**

In the present study, we performed 16S amplicon sequencing and metabolomic analyses of the rumen and hindgut microbiome of 8-month-old Dorper and Tan sheep, raised under identical feeding and management conditions, to explore the potential effects of gut microbiome and its metabolites on growth performance and meat quality.

**Results:**

Our study identified *Lactobacillus*, a marker genus in the rumen, to be significantly associated with the levels of fumaric acid, nicotinic acid, and 2-deoxyadenosine (*P*-value < 0.05). Statistical analysis showed that nicotinic acid was significantly negatively correlated with body weight (*P*-value < 0.01), while 2-deoxyadenosine was significantly positively correlated with fatty acids content (*P*-value < 0.05). There was a biologically significant negative correlation between *Phascolarctobacterium* and deoxycytidine levels in the hindgut. Deoxycytidine was significantly positively correlated with body weight, protein, and amino acid content. Differences in rumen fermentation patterns that are distinctive among breeds were identified. Tan sheep mainly used *Lactobacillus* and fumaric acid-mediated pyruvic acid for energy supply, while Dorper sheep utilize glycogenic amino acids. The difference of iron metabolism in the hindgut of Dorper sheep affects lipid production, while *Phascolarctobacterium* in Tan sheep is related to roughage tolerance. The accumulation of nucleosides promotes the growth performance of Dorper sheep.

**Conclusion:**

These findings provide insights into how the microbiome-metabolome-dependent mechanisms contribute to growth rate and fat contents in different breeds. This fundamental research is vital to identifying the dominant traits of breeds, improving growth rate and meat quality, and establishing principles for precision feeding.

## Introduction

Domestic animals provide human beings with abundant protein, an asset worth further exploitation by improving productivity; thus, studying factors that affect the growth and development of livestock is of great relevance (San et al., 1998; Kelley, 2006; Marcu et al., 2013; Park et al., 2018). One of the most important factors affecting productivity is the interaction between gut microorganisms and the host. Animals carry abundant and diverse communities of symbiotic microbes. Studies have shown that variation in the composition of commensal gut microbiome can lead to differences in nutrient absorption and utilization (Krajmalnik-Brown et al., 2012; Rowland et al., 2018). Gut microbes are involved in the regulation of many aspects of the body physiology and behavior, such as intestinal inflammatory response (Pittayanon et al., 2020) and mood (Kraimi et al., 2019). The rumen and hindgut of sheep are the most important fermentation sites in ruminants (Russell and Hespell, 1981). Gastrointestinal fermentation facilitates the digestion and absorption of fats, organic acids, and other substances (Spanghero et al., 2017; Schären et al., 2018); further, through the action of microorganisms, otherwise indigestible polysaccharides can be converted into energy by the host (Mackie, 2002). Current research shows that intestinal microorganisms play a role in animal feed efficiency, milk production, fat synthesis, and growth rate (Spanghero et al., 2017; Schären et al., 2018). Therefore, understanding the composition of gut microbes is crucial for deciphering breed traits.

Microbiome studies are often combined with metabolomes to explore the impact of microbes on host response (Couch et al., 2020; Ma et al., 2022). Microbial composition affects metabolite production and modification including short-chain fatty acids (SCFAs) (Wong et al., 2006), biogenic amines (Sudo, 2019), bile acids (Ramírez-Pérez et al., 2018), choline (Qiu et al., 2021), phenols (Espín et al., 2017). SCFA can modulate the immune response of the intestinal epithelium, and affects the growth of peripheral tissues (Parada Venegas et al., 2019). Bile acids participate in lipid metabolism and energy balance in the host through the enterohepatic circulation (Winston and Theriot, 2020). Furthermore, several food-derived metabolites, including curcumin (Han et al., 2021), the flavonoid quercetin (Wang et al., 2021), and whole grain starch (Li et al., 2021), have been shown to regulate lipid metabolism through the liver in mice. Indeed, gut metabolites act as messengers, sending signals to external tissues through the blood circulation (Mancini et al., 2018; Krautkramer et al., 2021). Therefore, it is evident that gut metabolite-related adaptations influence host performance.

In dairy cows, milk protein quality is related to rumen microbial composition. High abundance of members from the genus *Prevotella* is involved in amino acid and carbohydrate metabolism, ultimately improving production performance (Liu et al., 2022). Another study indicated that differences in hindgut microbes and metabolites in heifer calves affect residual feed intake (Elolimy et al., 2020). In sheep, bile acid supplementation reduces carcass and subcutaneous and tail fat deposition and modulates host fat distribution (Zhang et al., 2022). In addition, some studies have explored the rumen microbial composition and metabolic changes of Tibetan sheep (plateau breeds) at different growth stages (Li et al., 2020). The effects of different feeding regimens on host metabolism in Mongolian sheep have also been studied (Wang et al., 2020). However, the intestinal microbial composition and metabolites of animals in the same environment, diet, and life stage can be different, due to factors related to breed or adaptation (Lindsay et al., 2020; Ye et al., 2021). This effect of breed on gut microbiota and host performance under the same rearing conditions remains unclear. In order to effectively study the impact of gut microbiome and metabolome of sheep on the economic traits of sheep, we employed Dorper sheep, a South African composite breed that shows early maturity and rapid growth in the early stage, and Tan sheep, a fat-tailed sheep distributed in the Ningxia Hui Autonomous Region of China, with good meat quality and high intermuscular fat. Specifically, the aim of this study was to explore the functional interactions between the microbial composition and metabolites in the rumen and hindgut and host properties of Tan and Dorper sheep *via* microbiome and metabolomic analysis. The findings in this study will help to optimize feeding and health management of ruminants such as sheep and provide insights for analyzing factors that determine the dominant characteristics of breeds.

## Materials and methods

### Animals, sampling, and physiological parameters measurement

Considering that most lambs come to market when they become around 8 months old, Dorper (*n* = 8, D1–D4 represent male sheep and D5, D7, and D8 represent female sheep), and Tan sheep (*n* = 8, T1–T4 represent male sheep and T5–T8 represent female sheep) of this age were obtained from a commercial feedlot (Ningxia Autonomous Region, China) and used for experimentation. All sheep were maintained and fed under the standard livestock management practices, ensuring identical temperature and humidity, feed, grazing-housing time, and sufficient drinking water.

After assessing the phenotypic characteristics, the sheep were stunned using captive bolts and slaughtered by carotid exsanguination. Approximately 10 g of longissimus dorsi muscle tissue samples were collected and placed in a centrifuge tube with 4% paraformaldehyde to measure the physical properties of the muscle fibers and observe tissue sections. Simultaneously, raw meat samples were collected, trimmed into thin slices, and then stored at −20^°^C for meat quality determination. Fresh intestinal contents (about 4 ml) of Dorper and Tan sheep were collected from the rumen and middle of the cecum and colon for microbiome and metabolome analyses. Samples were placed in liquid nitrogen immediately after collection, and then transferred to a −80^°^C ultra-low temperature freezer for storage.

### DNA extraction, PCR amplification, and illumina NovaSeq sequencing

Microbial genome was extracted using TIAN amp Genomic DNA kit (TIANGEN Bio-Tek Co., Ltd., Beijing, China) according to the manufacturer’s instructions. The DNA was quantified by UV spectrophotometry (Thermo Fisher Scientific Inc., Waltham, MA, USA). The V3–V4 region of the 16 S rRNA gene was amplified using the following primers: ACTCCTACGGGAGGCAGCA (forward) and GGACTACHVGGGTWTCTAAT (reverse). AXYGEN gel recovery kit (Corning Bio-Tek Inc., Corning, NY, USA) was used for gel cutting and recovery, and fluorescence quantification of PCR amplification and recovery products was performed. Then, the samples were mixed proportionally according to the sequencing requirements of each sample. The sequencing library was prepared using TruSeq Nano DNA LT Library Prep Kit (Illumina BioTek Inc., San Diego, CA, USA). MiSeq sequencer (Illumina BioTek Inc.) was used to carry out 2 × 300 bp paired-end sequencing.

### Bioinformatics and statistical analyses

The raw data were subjected to qiime cutadapt trim-paired to excise the primer fragments of the sequence and discard the unmatched primer sequence; then the qiime dada2 denoise-paired of the DADA2 (2019.4) platform was used for quality control, denoising, splicing, and chimera generation for operational taxonomic units (OTUs)^1^ (Callahan et al., 2016; Bolyen et al., 2018). Singleton OTUs were removed after denoising (default sequence total is only one OTUs). Insertion and deletion errors in nucleic acid sequences were corrected using FrameBot (v1.2) software^2^. High-quality sequences were clustered at the 97% similarity level. Taxonomic classification and the OTU table output was performed using the SILVA classification database and (Callahan, 2018). The composition of microbial community at kingdom, phylum, class, order, family, genus, and species levels was obtained. Then, we used the Bray-Curtis distance algorithm to perform PCA and cluster dendrogram analysis, using the unweighted pair-group method to calculate the arithmetic means. LEfSe was used to conduct simultaneous differential analysis of all classification levels of microorganisms (Segata et al., 2011). Linear discriminant analysis (LDA) > 2 was used as the screening criterion. We selected the Random Forests algorithm for analysis to obtain important microorganisms (Breiman, 2001). PICRUSt2^3^ was used to predict 16 S rRNA gene sequences in MetaCyc^4^ and kyoto encyclopedia of genes and genomes (KEGG)^5^ databases to obtain differential pathway information and KEGG Orthology (Douglas et al., 2020).

Data between pair of groups were analyzed and visualized using the multiple t-test method of GraphPad Prism (version 8.0, San Diego, CA, USA). *P* < 0.05 was considered significant.

### Analysis of the rumen and hindgut metabolome

The metabolome of the rumen and hindgut was chromatographically separated using ultra-high performance liquid chromatography (UHPLC) and analyzed by mass spectrometry with a Q-Exactive high-resolution mass spectrometer (Thermo Fisher Scientific Inc.). The raw data collected by mass spectrometry was processed by Compound Discoverer 3.0 software (Thermo Fisher Scientific Inc.) for peak extraction, peak alignment, peak correction, normalization, and other data preprocessing. Output of a 3D data matrix consisting of sample names, peak information (including retention times and molecular weights), and peak areas was generated. Metabolite structures were identified by accurate mass matching (<25 ppm) and secondary spectrum matching. Data comparison was performed by searching HMDB, Metlin, and self-built databases (Personalbio Co., Ltd., Shanghai, China). Pattern recognition was performed using SIMCA-P 14.1 software (Umetrics Co., Ltd., Umea, Sweden), and the data were preprocessed by Pareto-scaling for analysis.

The online platform, MetaboAnalyst 5.0^6^ (Pang et al., 2021), was used for the PCA and multidimensional statistical based on targeted metabolites. MetOrigin^7^ was used to perform traceability analysis of metabolites and obtain differential metabolic pathways through KEGG data comparison. Sankey and network graphs were constructed to investigate biological and statistical correlations (Yu et al., 2022). Datasets for metabolites in the rumen and hindgut, and phenotype, were used for correlation analysis, and results were depicted in a heatmap (“pheatmap” package in R^8^).

## Results

### Characterization of phenotypes

**FIGURE 1:**
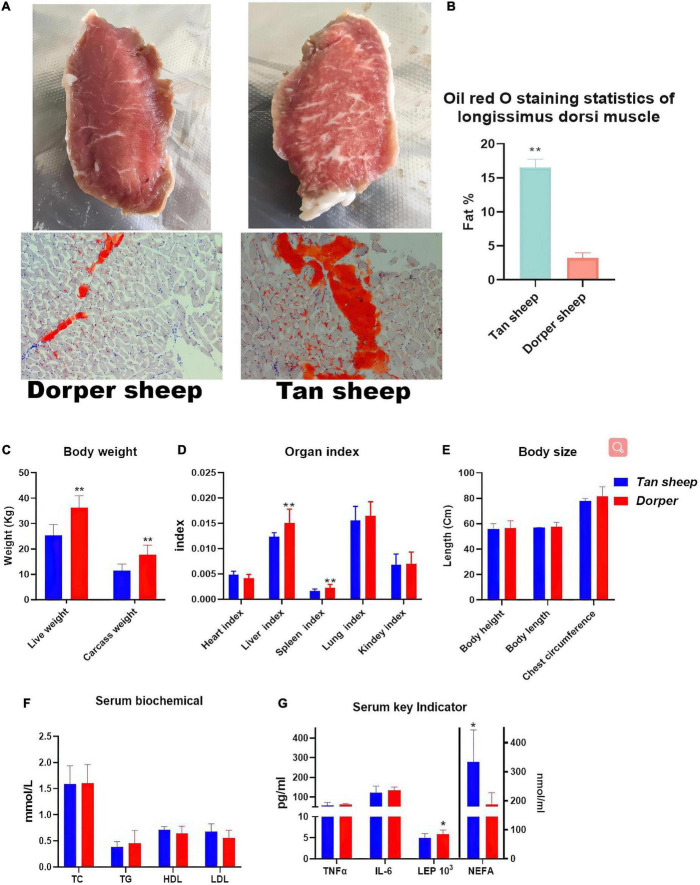
Phenotypic Trait Statistics. **(A)** The appearance map of the longest dorsal muscle of Dorper sheep (left) and Tan sheep (right) and the results of oil red O staining (bottom). **(B)** Image J statistics on Oil Red O staining results. (*n* = 6) **(C–E)** Statistical results of body weight, organ index and body size between breeds. **(F,G)** Statistical results of serum biochemistry and key indicator. *means *P-*value < 0.05, **means *P-*value < 0.01.

The body weight (including carcass weight) (*P* < 0.01, Figure 1C), liver and spleen organ index (*P* < 0.01, Figure 1D), and leptin hormone levels (*P* < 0.05, Figure 1G) of Dorper sheep were significantly higher than those of Tan sheep. The intramuscular fat content (*P* < 0.01, Figures 1A,B) and free fatty acid content (*P* < 0.05, Figure 1G) of Tan sheep were significantly higher than those of Dorper sheep. There was no significant difference in the body size and serum biochemical indices between the two groups (*P* > 0.05, Figures 1E,F). However, significant differences in muscle fiber size, protein content, partial fatty acid content, and selenium content between the two groups were noted (Supplementary File 1).

### Microbiome sequencing results and bacterial diversity

**FIGURE 2:**
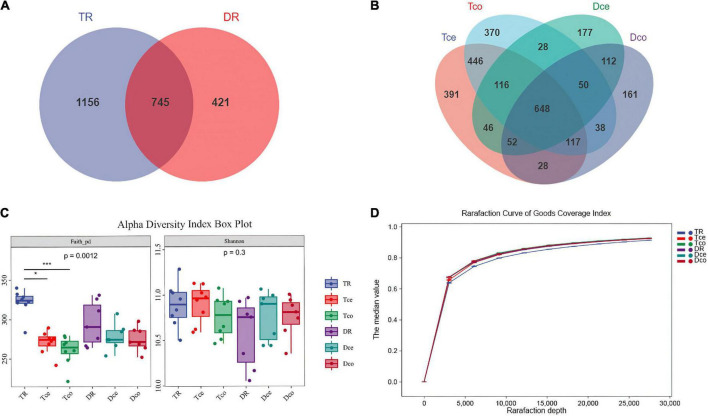
Sequencing results and statistical analysis of diversity. **(A)** Venn diagram showing the operational taxonomic units (OTUs) shared among the rumen. **(B)** Venn diagram showing the OTUs shared between the cecum-colon segments. **(C)** The Faith’s phylogenetic diversity (PD) and Shannon indices of six groups. **(D)** Rarefaction curve of Good’s coverage index. Each curve represents the mean within the group.

A total of 16 S rRNA sequencing of 45 samples generated 5,826,400 reads. After filtering the data, denoising, de-chimerism, and singleton checking, we obtained 2,508,854 clean reads. The number of OTUs shared by rumen segments was 745 (Figure 2A). The number of OTUs observed in the cecum and colon were 862 and 853, respectively (Figure 2B). The results showed that the number of common microorganisms in the rumen was lower than that in the cecum-colon.

The results of the Faith’s Phylogenetic Diversity index based on evolutionary characterization showed significant differences between the rumen and cecum-colon of Tan sheep (*P* < 0.001), while there was no difference between the rumen and cecum-colon of Dorper sheep, and no significant difference in the Shannon index among the groups (Figure 2C). The Good’s coverage index of each sample was more than 90%, and the curve tended to be flat, indicating that the sequencing data were sufficient to cover all bacterial communities (Figure 2D).

### Cluster analysis and microbial composition

**FIGURE 3:**
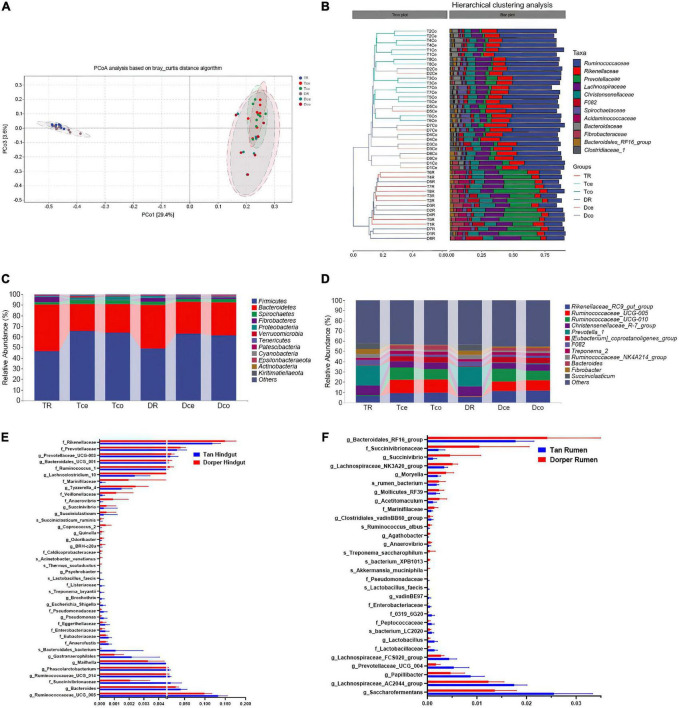
Microbial composition and ratio. **(A)** Principal coordinate analysis (PCoA) based on all samples. **(B)** The hierarchical tree shows the UPGMA clustering result. The abscissa indicates the distance between samples, the number after the group abbreviation represents the individual number, and the branch length indicates similarity. On the right is the stacked histogram of the top 10 abundant bacterial families in the sheep intestine. The abscissa indicates the proportion of bacteria. **(C)** The phylum-level microbial composition of each intestinal segment. **(D)** The genus-level microbial composition of each intestinal segment. **(E)** The ratio of key rumen microorganisms among breeds (*P-*value < 0.05). **(F)** The ratio of key hindgut microorganisms among breeds. (*P-*value < 0.05).

Principal component analysis (PCA) revealed that the rumen and cecum-colon were significantly separated, with 29.4 and 3.6% of the variation explained by principal component 1 (PC1) and PC3, respectively (Figure 3A). Permutational multivariate analysis of variance also showed that the microbiomes in the rumen and cecum-colon were significantly different between both breeds (adjusted *P* < 0.05), while no significant difference between the cecum and colon was found (adjusted *P* > 0.05) within each breed (Supplementary File 2). Since the microbial diversity and composition were similar in the cecum and colon, they were collectively referred to as the hindgut.

At the family level, the bacteria from the rumen and hindgut were grouped into two categories according to the hierarchical clustering results. Simultaneously, the similarity between the cecum and colon of the same host was higher than that of the same hindgut intestinal segment between hosts (Figure 3B). The microorganisms in the rumen and hindgut mainly include members from *Ruminococcaceae*, *Rikenellaceae*, *Prevotellaceae*, *Christensenellaceae*, and *Lachnospiraceae*. These bacteria accounted for approximately 70% of the gut microbes. *Prevotellaceae* and *Ruminococcaceae* were the dominant families in the rumen and hindgut, respectively. Firmicutes and Bacteroidetes were the two major groups of bacteria that constituted the gut microbiome, accounting for 90% of the total bacteria (Figure 3C). At the genus level, *Rikenellaceae_RC9_gut_group*, *Ruminococcaceae_UCG-005*, and *Ruminococcaceae_UCG-010* were the dominant genera in the hindgut, and their proportions were higher than those in the rumen (Figure 3D).

LEfSe analysis and random forest analysis were used to obtain dominant bacterial genera, and the intersection of the two results was obtained. We visualized 30 key bacteria in the rumen. Among them, *g_Saccharofermentans, g_Lachnospiraceae _AC2044_group* in Tan sheep, and *g_Bacteroidales_RF16_group* and *f_Succinivibrionaceae* in Dorper sheep were the dominant genera (LDA > 2, *P* < 0.05). In the random forest analysis, *f_Enterobacteriaceae*, *g_Lactobacillus*, and *s_bacterium_XPB1013* were the most important differential bacteria (Figure 3E).

In the hindgut, we visualized 40 bacteria at the intersection of the two analyses. *f_Rikenellaceae* and *g_Ruminococcaceae_UCG_005* were the most abundant differential bacterial genera (LDA > 2, *P* < 0.05; Figure 3F). Through joint analysis, we found that *f_Enterobacteriaceae, f_Pseudomonadaceae*, and *s_Lactobacillus_faecis* were highly abundant in the rumen and hindgut of Tan sheep, while *f_Marinifilaceae* and *g_Succinivibrio* were highly abundant in Dorper sheep.

### Microbial function prediction and intestinal metabolic pathways

Microbial functions are related to biosynthesis and metabolism; thus, the prediction of their function was performed *via* PICRUST2 using MetaCyc database as a reference. The abundance of biosynthetic processes was higher than that of other metabolic pathways. The main biological pathways included amino acid biosynthesis, nucleoside and nucleotide biosynthesis, cofactors, prosthetic groups, electron carriers, and vitamin biosynthesis (Supplementary File 3).

**FIGURE 4:**
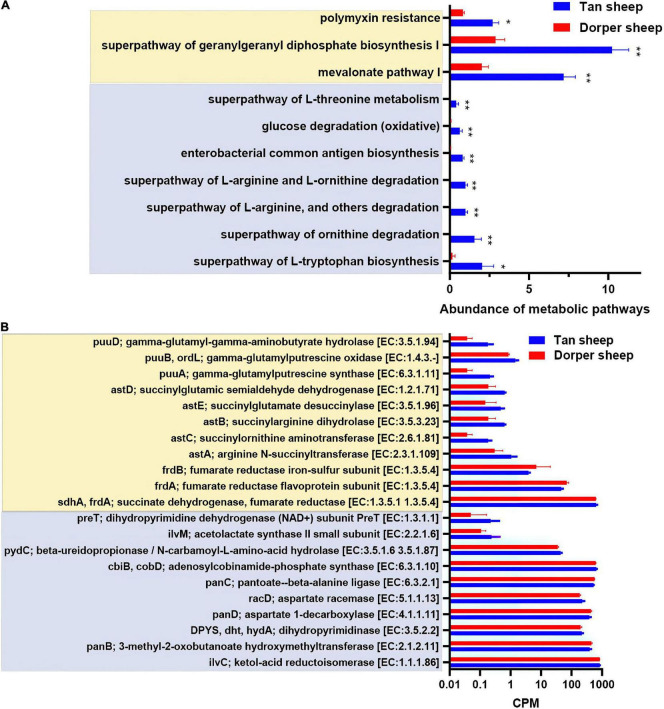
Predictive analysis and statistics of foregut and hindgut microbial function. **(A)** The differential analysis of metabolic pathways based on the MetaCyc database (*P-value < 0.05, **P-value < 0.01). **(B)** Critical metabolic enzyme enrichment based on the information of enzyme nomenclature (EC) functional orthologs in the kyoto encyclopedia of genes and genomes (KEGG) database (*P-*value < 0.05). (Light yellow indicates the rumen and light blue indicates the hindgut).

The results of metabolic pathways showed that mevalonate pathway I, a super pathway of geranylgeranyl diphosphate biosynthesis I (*via* mevalonate), polymyxin resistance was more active in the rumen of Tan sheep. In contrast, the hindgut mainly included seven differential metabolic pathways, including the super-pathway of L-threonine metabolism (Figure 4A).

In the KEGG orthologs database, we screened 1,194 differential orthologs in the rumen. Differential orthologs (2,098) were screened in the hindgut (*P* < 0.05). We visualized 21 differential metabolic orthologs in the rumen and hindgut (Figure 4B). The M00879 (arginine succinyl transferase pathway, astA, astB, astC, astD, and astE), and M00136 (GABA biosynthesis, puuA, puuB, and puuD) reaction modules in the rumen were highly expressed in Tan sheep. Additionally, M00019 (valine/isoleucine biosynthesis, ilvC, and ilvM) and M00046 (pyrimidine degradation, DPY S, pydC, and preT) in the hindgut were more abundant in Tan sheep, and M00119 (pantothenate biosynthesis, panB, panC, and panD) was more active in Dorper sheep.

### Profiles of rumen and hindgut metabolome

In the metabolomic LC-MS analysis, 1,193 compounds were identified in positive and negative ion modes. After *t*-test and variable importance in projection (VIP) filtering, 128 metabolites were found to be significantly different between the two groups: 51 compounds were significantly higher in Dorper sheep, and 77 compounds were significantly higher in Tan sheep (*P* < 0.05, VIP > 1). KEGG database analysis revealed the annotation information of 73 metabolites (Supplementary File 4).

**FIGURE 5:**
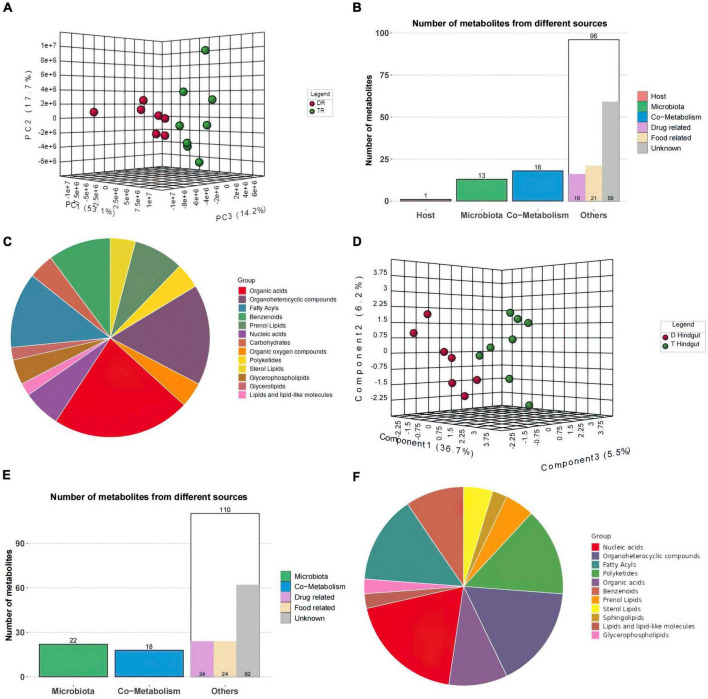
Metabolome Statistics Analysis **(A,D)** Three-dimensional principal component analysis of the rumen and hindgut metabolome. **(B,E)** Classification of rumen and hindgut differential metabolite sources according to host source, microbial source and common source. **(C,F)** Chemical structure analysis of rumen and hindgut differential metabolites according to super class.

The PCA results showed significant separation of rumen compounds between the different groups, with 53.1, 17.7, and 14.2% of the variation explained by principal component 1 (PC1), PC2, and PC3, respectively (Figure 5A). One host-specific and 13 microbiota-specific metabolites were identified in rumen. Moreover, 16 drug and 21 food-related metabolites were identified (Figure 5B), while organic acids, organoheterocyclic compounds, and fatty acyls were the main components in rumen (Figure 5C).

In hindgut, 150 significantly different compounds were observed. KEGG annotated information for 111 metabolites (Supplementary File 4). Forty-two metabolites were significantly elevated in the hindgut of Tan sheep, while 108 were highly expressed in the hindgut of Dorper sheep.

PC1, PC2, and PC3 explained 36.7, 6.2, and 5.5% of the variations, respectively in hindgut (Figure 5D). Twenty-two metabolites were derived from microorganisms and related to drugs. Eighteen metabolites were co-metabolized by the host and microbes, 24 microbes were food-associated and drug-related, and 62 metabolites were of unclassified origin (Figure 5E). Nucleic acids, organoheterocyclic compounds, and fatty acyls were the main metabolites in hindgut (Figure 5F).

Based on databases of organ tissues, analysis showed that myelin tissue was enriched in the rumen, while neural tissue was enriched in the hindgut.

### Function analysis of rumen and hindgut metabolome

**FIGURE 6:**
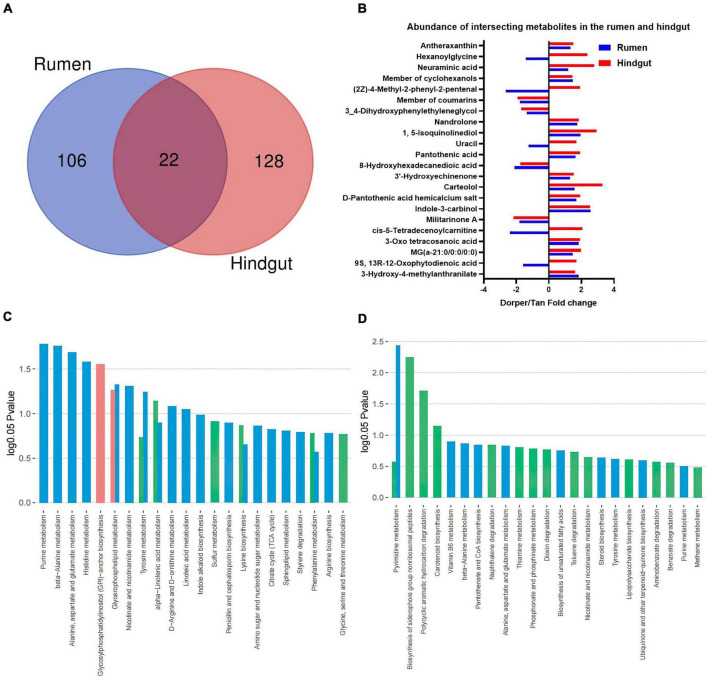
Functional analysis of the metabolome **(A)** Venn analysis of significantly different metabolites in rumen and hindgut. **(B)** Visualization of key metabolites in the rumen and hindgut. Dorper/Tan foldchange is +. **(C,D)** KEGG enrichment analysis of rumen and hindgut differential metabolites. Venn diagram represents the statistics of enriched metabolic pathways in host-microbe-co-metabolism.

Venn enrichment revealed the presence of unique metabolites in the rumen and hindgut. Twenty-two metabolites were differentially expressed in different intestinal segments (Figure 6A). The expression patterns of intersection metabolites, mainly including organic heterocyclic compounds, benzenes, and fatty acyl groups, related to β-alanine metabolism and fatty acid oxidation processes, were visualized (Figure 6B).

The KEGG enrichment analysis showed 11 and four differential metabolic pathways enriched in the rumen and hindgut, respectively (*P* < 0.05). In rumen, glycosylphospatidylinositol (GPI)-anchor biosynthesis metabolism is unique to the host, and its related lipid metabolism includes (alpha)-linolenic acid and glycerophospholipid metabolism. Furthermore, we identified some amino acid-related metabolic pathways, including alanine, aspartate, and glutamate metabolic pathways. This also included purine, histidine, nicotinate, and nicotinamide metabolism which were significantly different metabolic processes (Figure 6C).

In hindgut, biosynthesis of siderophore group non-ribosomal peptides, polycyclic aromatic hydrocarbon degradation, and carotenoid biosynthesis are metabolic processes specific to microorganisms. Among the co-metabolism processes, pyrimidine metabolism is significantly different (Figure 6D).

### Relationship between phenotype, microbiome, and metabolome

**FIGURE 7:**
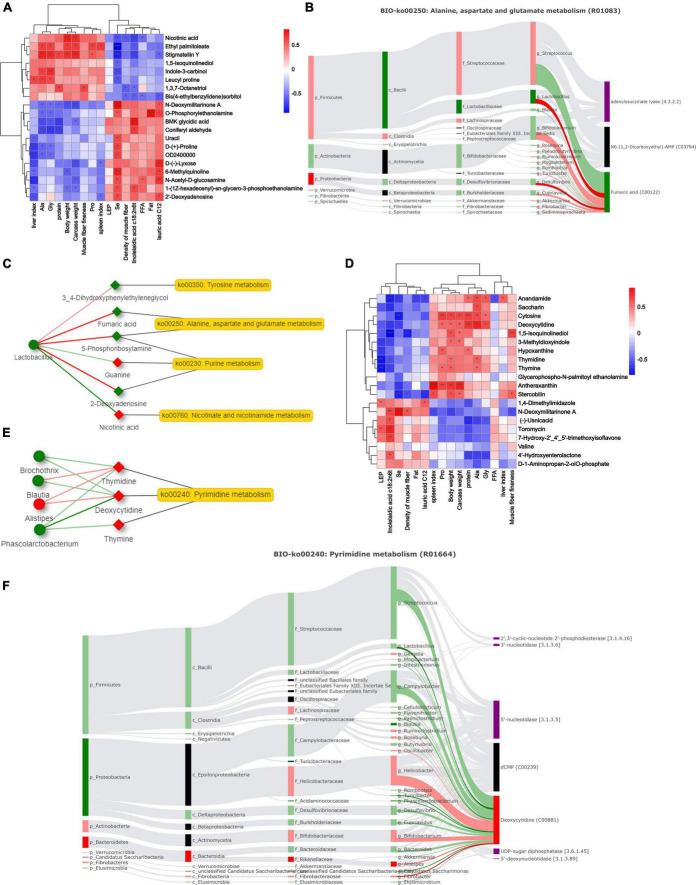
Correlation analysis of microbes, metabolites and phenotypes. **(A,D)** Differential phenotypes among breeds and Spearman correlations of rumen and hindgut metabolites (*P-value < 0.05, **P-value < 0.01, ***P-value < 0.001). **(B,F)** Biologically relevant sankey diagrams of microorganisms and metabolites in rumen and hindgut. Red indicates high expression in Dorper sheep, and green indicates high expression in Tan sheep (*P-*value < 0.05). The light-colored line indicates that the spearman correlation is not significant (*P-*value > 0.05), and the dark line indicates that the spearman correlation is significant (*P-*value < 0.05). **(C,E)** Network analysis of key microbiota and metabolites in rumen and hindgut.

Spearman’s rank correlation analysis revealed 20 significant correlations between rumen microbiota and phenotypes (*R* > 0.5, *P* < 0.05; Figure 7A). The weight-related indices showed the same trend, significantly positively correlated with nicotinic acid and stigmatellin Y. Furthermore, *N*-deoxymilitarinone A and *O*-phosphorylethanolamine showed significant positive correlations with meat quality-related traits. Fatty acids, selenium trace element, and intramuscular fat content had similar correlations. We further conducted Spearman correlation analysis between metabolites and microbes. *Lactobacillaceae*, *Succinivibrionacea*, *Lactobacillus*, and *Saccharofermentans* exhibited a strong correlation with metabolites (Supplementary File 5). Traceability analysis revealed biological and statistical correlations between *Lactobacillus* and the six metabolites including fumaric acid (R01083) (Figure 7B). A network diagram showing interactions between rumen microbe-metabolite pathways is presented in Figure 7C. The correlation between *Lactobacillus*, nicotinic acid, and 2-deoxyadenosine was significantly associated with the phenotype in a previous analysis (*P* < 0.05). The enriched niacin and nicotinamide metabolism, and purine metabolism exhibited significant differences.

The correlation between metabolites and phenotypes in the hindgut was lower than that in the rumen (Figure 7D). At the genus level, we found a correlation between 23 bacteria and their metabolites, including *Succinivibrio* and *Phascolarctobacterium*. Figure 7F shows the Sankey correlation diagram in pyrimidine metabolism. The hindgut microbial network analysis showed that *Phascolarctobacteri*um and deoxycytidine were significantly negatively correlated (*P* < 0.05, Figure 7E). Thymidine and deoxycytidine were highly expressed in Dorper sheep and significantly enriched in the pyrimidine metabolic pathway.

### Metabolome and microbiome diagram construction based on relationship networks

**FIGURE 8:**
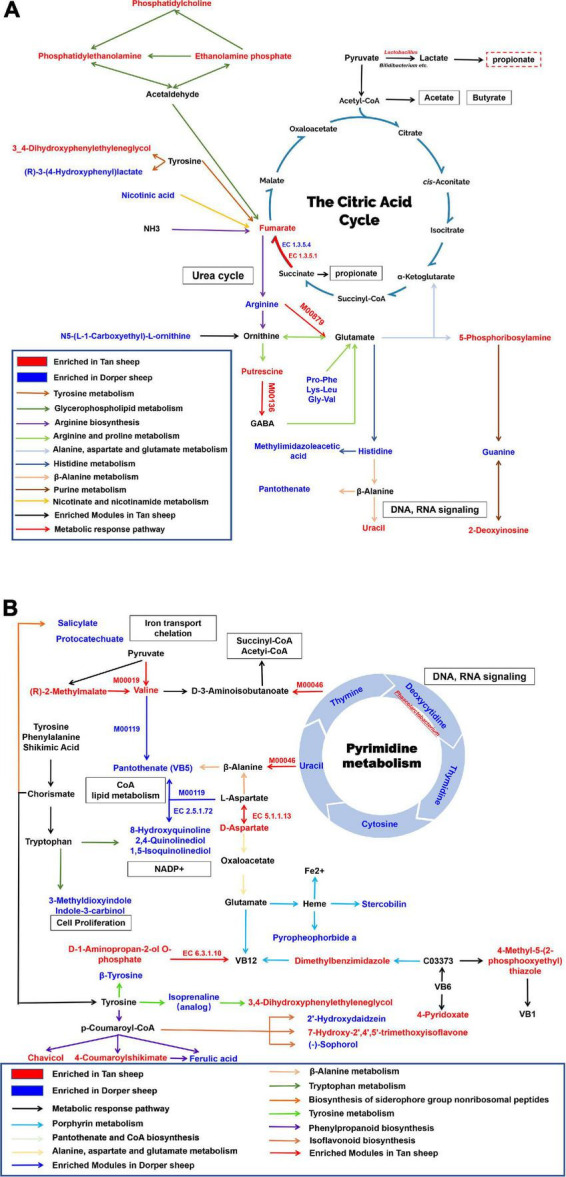
Rumen and hindgut biological metabolic networks. **(A)** Rumen metabolic network. **(B)** Hindgut metabolic network. EC denotes enzymes for KEGG enrichment analysis. M represents a module in a metabolic pathway. Red represents metabolites abundant in Tan sheep, blue represents metabolites abundant in Dorper sheep, and black represents metabolites with no significant difference. Squares indicate involved biological processes. The enriched metabolites were *P-*value < 0.05, VIP > 1.

We constructed a metabolic network map of metabolites present in rumen. Nine metabolic pathways were identified, including tyrosine and purine metabolic pathways. The M00879 module (arginine succinyl transferase pathway, arginine to glutamate) and the M00136 module (GABA biosynthesis, prokaryotes, putrescine to GABA) were significantly enriched in Tan sheep (*P* < 0.05). enzyme nomenclature (EC) 1.3.5.1 (succinate dehydrogenase/fumarate reductase) was significantly enriched in Tan sheep and EC1.3.5.4 (fumarate reductase flavoprotein subunit) was significantly enriched in Dorper sheep (*P* < 0.05). The biological processes related to the rumen included VFA production, urea cycle, and DNA, and RNA signaling (Figure 8A).

The hindgut was enriched in nine metabolic pathways, including that of pyrimidine and porphyrin. Overlapping metabolites between the pathways were connected as nodes. The results of the microbial analysis showed that M00046 (pyrimidine degradation, uracil to beta-alanine, thymine to 3-aminoisobutanoate) and M00019 (valine/isoleucine biosynthesis, pyruvate to valine/2-oxobutanoate to isoleucine) were significantly enriched in Tan sheep. M00119 (pantothenate biosynthesis, valine/l-aspartate to pantothenate) was significantly enriched in Dorper sheep, whereas EC 5.1.1.13 and EC 6.3.1.10 were significantly enriched in Tan sheep (*P* < 0.05). Biological processes in the hindgut involved iron transport, flavonoid metabolism, and DNA, and RNA signaling (Figure 8B).

## Discussion

By integrating analysis of the microbiome and metabolome of the rumen and hindgut, we identified microbe-dependent and host-metabolism-dependent mechanisms that lead to differences in growth and meat quality and constructed a network of microbial composition, function, and metabolites that differentiate these traits.

Differences in bacterial ratios between breeds is intriguing. In the hindgut, the proportion of Firmicutes and Bacteroidetes in Tan sheep was higher (2.4%) than that in Dorper sheep (1.05%), and this proportion was positively correlated with obesity (Ley et al., 2005). The lower abundance of members from *Rikenellaceae* has also been implicated in an increased fat content in Tan sheep (Arnoriaga-Rodríguez et al., 2020). *Lachnospiraceae* colonization experiments show that it can cause a significant increase in the weight of internal organs (such as the liver) and reduce plasma insulin levels (Kameyama and Itoh, 2014). These bacterial differences contributed to the sheep phenotype and are in agreement with previous studies. *Saccharofermentans* are believed to be associated with indigestible high-lignin dietary components (Langda et al., 2020) and can convert succinic acid into acetic and propionic acids (Li et al., 2012; La Reau and Suen, 2018). Members from *Succinivibrionaceae*, the dominant bacteria in the rumen of Dorper sheep identified in this study, can produce succinic acid (Fernández-Veledo and Vendrell, 2019). Interestingly, in this study, the identified succinate was not significantly enriched. This may be because *Succinivibrionaceae* are also involved in other processes such as starch, hemicellulose, and xylan degradation, and not just succinic acid production (Bryant, 1970). *Lactobacillus* are “good bacteria,” which are important both for maintaining homeostasis and producing lactic acid for energy (Turroni et al., 2014). The genus *Phascolarctobacterium* is a group of beneficial bacteria capable of producing short chain fatty acids (Wu et al., 2017). This valuable substrate can provide sufficient raw materials for fat synthesis (Ladeira et al., 2018). The changes in the abundances of *Lactobacillus* and *Phascolarctobacterium* are indicative of the strong adaptability of Tan sheep; however, the beneficial effects of these genera in sheep need further exploration.

Energy utilization in the rumen and lipid metabolism in the hindgut are notable features that vary among different breeds. Fumaric acid in Tan sheep increased the production of rumen propionic acid, promoted gluconeogenesis, and improved feed utilization (Li et al., 2018). Dorper sheep were enriched in glycogenic amino acids and their derivatives, indicating that they mainly provided energy in the form of amino acids (D’Andrea, 2000). Therefore, we speculate rumen fermentation patterns among breeds can be different. The hindgut metabolic network revealed differences in porphyrin and iron metabolism. Iron is involved in lipid metabolism and can promote fatty acid import and lipid droplet formation through synergistic action in the liver (Anderson and Shah, 2013; Rockfield et al., 2018). Fatty acids such as *N*-palmitoylserinol and 2-Oxo non-adecanoic acid were higher in Dorper sheep, indicating that differences in fat metabolism serve as specific characteristic among different breeds.

Niacin and pyrimidine were found to be involved in the regulation of body functions, as revealed by the microbe-metabolite-phenotype network. Specifically, correlation analysis of the metabolic phenotypes showed that niacin was positively and negatively correlated with body weight and fat content, respectively. As a precursor of NAD + and NADP +, niacin is important for catabolic and anabolic redox reactions. Additionally, niacin is known for its antilipolytic action *via* a hydroxycarboxylic acid-2-receptor-dependent mechanism (Chen et al., 2019). Niacin supplementation may help lower the plasma triglyceride and non-esterified fatty acid concentrations (Hristovska et al., 2017; Wei et al., 2018). Differences in pyrimidine metabolism are prominent features of the hindgut while different nucleoside combinations can affect animal growth performance (Daneshmand et al., 2017b). In broiler studies, it was found that feeding with yeast extract as a source of nucleotides did not affect animal growth parameters (Jung and Batal, 2012), whereas in piglets, nucleotides could beneficially influence body weight, body weight gain, and feed intake (Superchi et al., 2012). Our findings are consistent with the latter case. Different inherent, management, and environmental factors, such as animal species, age, and feeding conditions, may explain this inconsistency observed by different researchers. In addition, some studies have pointed out that since the main components of the nucleoside *de novo* pathway are various amino acids, such as glutamine, aspartic acid and glycine, the accumulation of exogenous nucleosides in epithelial cells can cause amino acid retention (Berens et al., 1995; Daneshmand et al., 2017a). Higher levels of amino acids were detected in the intestinal tract of Dorper sheep than in the intestinal tract of Tan sheep under the same feeding environment; this indicates that this difference may be determined by breed characteristics; however, this claim needs to be further verified.

Some metabolites were not annotated in detail in the KEGG database. *N*-deoxymilitarinone A, a pyridone alkaloid and a natural product found in *Cordyceps farinosa*, possibly playing a role in the nervous system through the brain-gut axis (Cheng et al., 2006), was significantly positively correlated with selenium content. Stigmatellin Y, a chromone inhibitor that inhibits respiratory complex I at high concentrations (Von Jagow and Ohnishi, 1985), was negatively and positively correlated with meat quality and growth traits, respectively. Anandamide, a fatty acid neurotransmitter, acts on cannabinoid receptors in the nervous system and regulates feeding behavior and pleasure (Pacher et al., 2006). Studies have shown that chrysophanol can effectively inhibit lipid accumulation *in vitro*, reduce body weight in mice, and improve insulin sensitivity (Liu et al., 2020). The biological roles of these substances in sheep require further investigation.

Since evidence for a direct contribution microbial functions is lacking and metabolome heritability is currently poorly understood, future studies are needed to assess the causal relationship between these functions and metabolic elements. Our findings provide insights highlighting the possibility of controlling microbial functions and metabolites through genetic selection.

## Conclusion

Our study identified differences in rumen fermentation patterns that are distinctive among breeds. Tan sheep mainly use *Lactobacillus* and fumaric acid-mediated pyruvic acid for energy supply, while Dorper sheep mainly utilize glycogenic amino acids. The difference of iron metabolism in the hindgut of Dorper sheep affects lipid production, while *Phascolarctobacterium* in Tan sheep is related to roughage tolerance. The accumulation of nucleosides promotes the growth performance of Dorper sheep. Overall, we obtained statistical and biological correlations between microbiome and metabolite-related parameters and sheep phenotype. Gut microbiome- and metabolite-dependent mechanisms investigated in the present study provide new insights into the connection of altering the microbiota-metabolites for enhancing breed traits.

## Data availability statement

The rumen and hindgut 16S amplicon sequences were deposited in the NCBI Sequence Read Archive (SRA) under the accession number: PRJNA857330.

## Ethics statement

This animal study was reviewed and approved by the Animal Care and Use Committee of China Agricultural University (permit number: SKLAB-2012-04-07).

## Author contributions

YM contributed to ranch sampling, complete analysis, and writing the manuscript. XY provided analytical ideas and contributed to branch sampling. GH, XiD, and TX were mainly involved in pasture sampling. XL and DF participated in pasture sampling and assisted with experimental sample processing. XuD contributed to experimental conception, ranch work arrangement, and manuscript modifications. All authors have read and agreed to the published version of the manuscript.

## Footnotes

1
https://qiime2.org/

2
https://github.com/rdpstaff/Framebot

3
https://github.com/picrust/picrust2/releases

4
https://metacyc.org

5
https://www.kegg.jp/

6
https://www.metaboanalyst.ca/

7
http://metorigin.met-bioinformatics.cn/home/

8
https://www.r-project.org
