# Supplementary material for: Contribution of gut microbiomes and their metabolomes to the performance of Dorper and Tan sheep
Source: Front Microbiol. 2022 Nov 28;13:1047744. doi: 10.3389/fmicb.2022.1047744 (PMC9742522; doi:10.3389/fmicb.2022.1047744)
Supplement: Supplementary file 1 [file Table_1.pdf]

Table S1 Physiological parameters of Tan sheep and Dorper sheep.

Items	Mean Tan (n=8)	Mean Dorper (n=8)	SEM	*P*
**Body, organ Performance**				
Body Weight (kg)	25.36	36.28	2.23	< 0.01**
Body height (cm)	56.38	58.75	2.67	0.38
Body length(cm)	55.13	57.75	1.84	0.18
Chest circumference(cm)	77.38	83.00	2.84	0.32
Carcass weight(kg)	11.75	17.75	1.58	< 0.01**
Backfat(cm)	0.40	0.32	0.03	0.33
GR	0.79	1.04	0.16	0.14
Heart index	0.48	0.41	0.04	0.08
Liver index	1.24	1.51	0.10	0.02*
Spleen index	0.16	0.23	0.02	0.02*
Lung index	1.56	1.65	0.14	0.52
Kidney index	0.68	0.70	0.11	0.83
**Meat Performance**				
PH	5.36	5.70	0.18	0.08
Shear force (N)	21.42	26.35	4.63	0.31
Muscle fiber fineness(μm)	27.38	36.51	4.24	0.05*
Density of muscle fiber (N/mm^2)	921.6	568.1	100.4	< 0.01**
Cooked Meat Percentage (%)	59.65	62.49	2.77	0.32
Driage (%)	32,13	35.73	2.49	0.17
Fat (%)	5.43	3.41	0.93	0.05*
Protein (%)	20.46	21.92	0,54	0.02*
Creatinine (μg/g)	366.2	606.3	88.84	0.02*
Inosinic acid (μg/g)	1902	1400	243	0.06
Cholesterol (μg/g)	338.8	306.6	27.34	0.26
**Meat mineral Performance**				
Cu（mg/kg)	1.52	1.39	0.26	0.64
Zn（mg/kg)	24.58	26.38	2.08	0.40
Se（mg/kg)	301.2	141.1	42.86	< 0.01**
Ca（mg/kg)	155.3	139.0	13.38	0.24
Fe（mg/kg)	22.87	24.84	1.97	0.33
Mg（mg/kg)	282.6	300.1	22.12	0.44
**Meat fatty acid Performance**				
**Saturated fatty acid**				
**∑ Saturated fatty acid**	219.9	146.5	24.34	< 0.01**
Butyric acid C4 (μg/g)	19.43	19.55	2.75	0.96
Caproic acid C6 (μg/g)	13.09	22.68	9.37	0.32
Capric acid C10 (μg/g)	9.15	4.01	2.50	0.06
Undecanoic acid C11 (μg/g)	219.6	197.9	19.99	0.30

Lauric acid C12 (μg/g)	9.59	2.99	2.92	0.04*
Tridecanoic acid C13 (μg/g)	2.58	2.51	0.73	0.93
Myristic acid C14 (μg/g)	153.3	77.95	41.58	0.09
Pentadecanoic acid C15 (μg/g)	19.99	16.86	5.96	0.61
Palmitic acid C16 (μg/g)	1316.0	818.8	301.0	0.12
Heptadecanoic acid C17 (μg/g)	59.14	53.36	17.76	0.75
Stearic acid C18 (μg/g)	1029.0	685.0	220.8	0.14
Eicosanic acid C20 (μg/g)	1.91	0.81	0.98	0.28
Behenic acid C22 (μg/g)	5.46	1.90	1.79	0.07
**Monounsaturated fatty acids**				
∑ Monounsaturated fatty acid	660.2	303.9	76.15	0.04*
C14:1 (μg/g)	7.74	4.02	2.12	0.10
C16:1 (μg/g)	94.04	61.21	24.26	0.20
C17:1 (μg/g)	31.58	26.05	8.69	0.53
Elaidic acid C18:1n9t (μg/g)	79.06	80.78	34.28	0.96
Oleinic acid C18:1n9c (μg/g)	2089	1348	464.4	0.13
**Polyunsaturated fatty acids**				
∑ Polyunsaturated fatty acids	87.01	75.45	6.45	0.08
Linolelaidic acid c18:2n6t (μg/g)	9.08	1.83	2.07	< 0.01**
Linoleic acid c18:2n6c (μg/g)	195.9	169.8	35.34	0.47
Arachidonic acid c20:4n6 (μg/g)	56.03	54.74	5.70	0.82
**Meat amino acid Performance**				
Aspartic acid	1.73	2.07	0.17	0.06
Threonine	0.94	1.08	0.09	0.14
Serine	0.81	0.97	0.08	0.05
Glutamic acid	2.86	3.54	0.29	0.04*
Glycine	0.85	1.05	0.09	0.06
Alanine	1.23	1.51	0.10	0.02*
Cysteine	0.09	0.01	0.04	0.07
Valine	1.05	1.38	0.16	0.06
Methionine	0.53	0.89	0.20	0.10
Isoleucine	0.98	1.29	0.18	0.10
Leucine	1.70	2.09	0.21	0.09
Tyrosine	0.72	0.78	0.10	0.58
Phenylalanine	1.39	1.33	0.10	0.56
Lysine	2.07	2.19	0.16	0.47
Histidine	1.07	0.87	0.10	0.06
Arginine	1.19	1.27	0.09	0.39
Proline	0.83	1.03	0.09	0.04*
**Serum biochemical Performance**				
TC (mmol/L)	1.59	1.60	0.18	0.93
TG (mmol/L)	0.39	0.46	0.09	0.46
HDL (mmol/L)	0.71	0.65	0.05	0.24
LDL (mmol/L)	0.68	0.56	0.07	0.13

TNFα（pg/ml)	57.39	61.49	3.91	0.30
IL-6（pg/ml)	122.8	133.9	9.03	0.23
LEP（ng/ml)	4.95	5.84	0.36	0.02*
NEFA (nmol/ml)	244.8	62.96	72.18	0.03*

SEM, stand error of the mean. *P* Values between Tan sheep and Dorper sheep were calculated using t test.
