# Supplementary material for: Contribution of gut microbiomes and their metabolomes to the performance of Dorper and Tan sheep
Source: Front Microbiol. 2022 Nov 28;13:1047744. doi: 10.3389/fmicb.2022.1047744 (PMC9742522; doi:10.3389/fmicb.2022.1047744)
Supplement: Supplementary file 2 [file Table_2.pdf]

S2 Permutational multivariate analysis of variance in the intestinal

Group1	Group2	Samplesize	Permutations	pseudoF	pvalue	qvalue
all	-	45	999	4.423118	0.001	-
TR	Tce	16	999	8.691055	0.001	0.0025
TR	Tco	16	999	8.619714	0.001	0.0025
TR	DR	15	999	1.410882	0.009	0.01125
TR	Dce	15	999	7.375679	0.001	0.0025
TR	Dco	15	999	7.393986	0.001	0.0025
Tce	Tco	16	999	0.380703	0.982	0.982
Tce	DR	15	999	7.440696	0.002	0.00375
Tce	Dce	15	999	1.230247	0.008	0.010909091
Tce	Dco	15	999	1.309371	0.001	0.0025
Tco	DR	15	999	7.361378	0.001	0.0025
Tco	Dce	15	999	1.216327	0.006	0.009
Tco	Dco	15	999	1.186606	0.013	0.015
DR	Dce	14	999	6.268908	0.003	0.005
DR	Dco	14	999	6.280443	0.002	0.00375
Dce	Dco	14	999	0.304234	0.964	0.982
