# Supplementary material for: Contribution of gut microbiomes and their metabolomes to the performance of Dorper and Tan sheep
Source: Front Microbiol. 2022 Nov 28;13:1047744. doi: 10.3389/fmicb.2022.1047744 (PMC9742522; doi:10.3389/fmicb.2022.1047744)
Supplement: Supplementary file 3 [file Table_3.pdf]

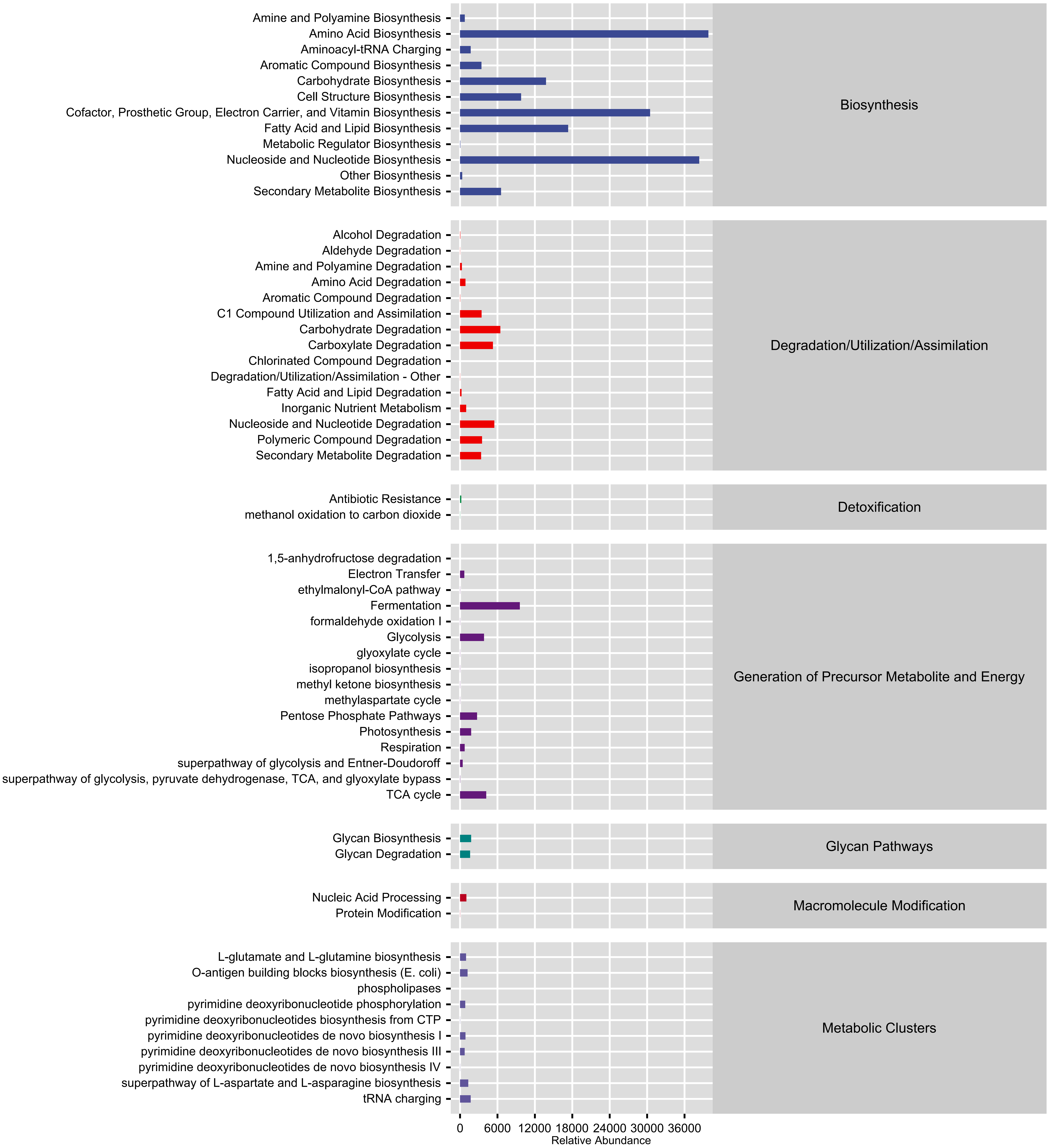

Fig.S3 Predictive analysis and statistics of rumen and hindgut microbial function.
The abundance of the differential metabolic pathways based on the MetaCyc database. The abscissa is the abundance count of the classification, the ordinate is the functional pathway of MetaCyc's second classification level, and the rightmost is the first-level classification to which this pathway belongs.
