# Supplementary figures and images for: Contribution of gut microbiomes and their metabolomes to the performance of Dorper and Tan sheep
Source: Front Microbiol. 2022 Nov 28;13:1047744. doi: 10.3389/fmicb.2022.1047744 (PMC9742522; doi:10.3389/fmicb.2022.1047744)

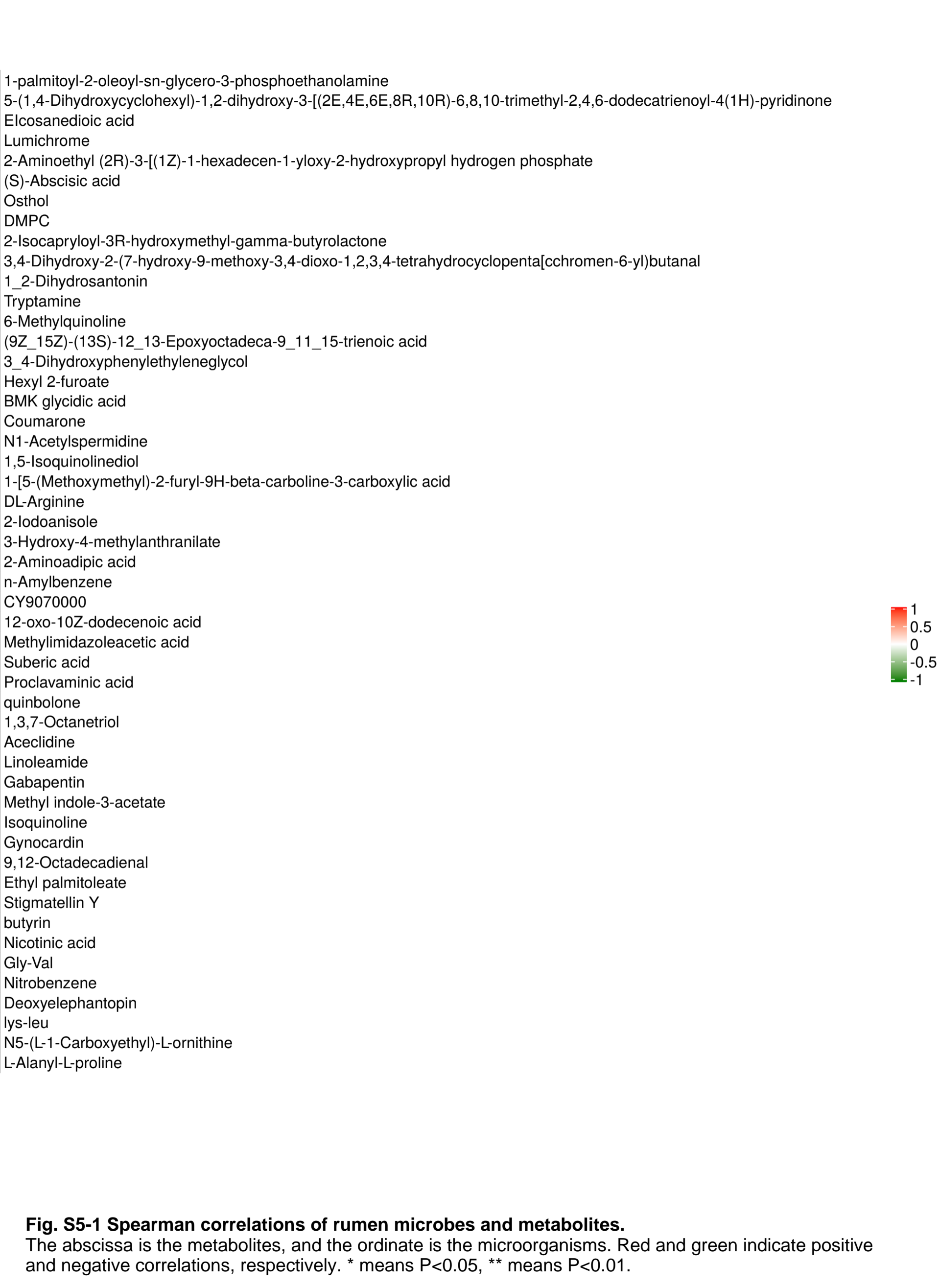

Supplement: Supplementary file 5 [file Table_5.pdf]
